# Renal cell carcinoma lung metastases treated by radiofrequency ablation integrated with systemic treatments: over 10 years of experience

**DOI:** 10.1186/s12885-019-6345-2

**Published:** 2019-12-03

**Authors:** Alexis Gonnet, Laura Salabert, Guilhem Roubaud, Vittorio Catena, Véronique Brouste, Xavier Buy, Marine Gross Goupil, Alain Ravaud, Jean Palussière

**Affiliations:** 1 Department of Radiology, Institut Bergonié, 229 cours de l’Argonne, 33000 Bordeaux, France; 2 Department of Radiology CH Pau, 4 Boulevard Hauterive, 64000 Pau, France; 3 Department of Medical Oncology, Hospital Saint-Andre, Univ. Bordeaux, 33000 Bordeaux, France; 4 Department of Medical Oncology, Institut Bergonié, 33000 Bordeaux, France; 5 Department of Statistics, Institut Bergonié, 33000 Bordeaux, France

**Keywords:** Renal cell carcinoma, Metastases, Lung metastases, Radiofrequency ablation, Local treatment, Oligometastasis

## Abstract

**Background:**

To determine safety and efficacy of radiofrequency ablation (RFA) for local treatment of lung metastases of renal cell carcinoma (RCC), sequenced or combined with systemic treatments.

**Methods:**

Retrospectively, we studied 53 patients treated by RFA for a maximum of six lung metastases of RCC. The endpoints were local efficacy, overall (OS), disease-free (DFS), pulmonary progression-free (PPFS) and systemic treatment-free (STFS) survivals, complications graded by the CTCAE classification and factors associated with survivals. Potential factors analysed were: clinical and pathological data, tumoral staging of TNM classification, primary tumor histology, Fuhrman’s grade, age, number and size of lung metastases and extra-pulmonary metastases pre-RFA.

**Results:**

One hundred metastases were treated by RFA. Median follow-up time was 61 months (interquartile range 90–34). Five-year OS was 62% (95% confidence interval (CI): 44–75). Median DFS was 9.9 months (95% CI: 6–16). PPFS at 1 and 3 years was 58.9% (95%CI: 44.1–70.9) and 35.2% (95%CI: 21.6–49.1), respectively. We observed 3% major complications (grade 3 and 4 of CTCAE classification). Local efficacy was 91%. Median STFS was 28.3 months. Thirteen patients (25%) with lung recurrence could be treated by another RFA. T3/T4 tumors had significantly worse OS, PPFS and STFS. Having two or more lung metastases increased the risk of pulmonary progression more than threefold.

**Conclusion:**

Integrated to systemic treatment strategy, RFA is safe and effective for the treatment strategy of lung metastasis from RCC with good OS and long systemic treatment-free survival. RFA offers the possibility of repeat procedures, with low morbidity.

## Background

Renal cell carcinoma (RCC) is the seventh most common solid cancer, with an incidence of 14.5 per 100,000 people [1, 2]. The most frequent site of metastasis and recurrence is the lung, with more than 50–60% of patients developing lung metastases [3]. Recently, both targeted therapy and immunotherapy have broadened treatment options and improved prognosis [4–6], but systemic treatments remain expensive and targeted therapies are known to affect quality of life by their related toxicities.

Percutaneous image-guided thermal ablation is increasingly used to treat metastatic disease. Thermal ablation like radiofrequency (RFA) is effective with few complications and repeatable without distorting lung function [7]. RFA for the treatment of RCC lung metastases and repeat RFA have rarely been reported [8, 9]. As a local treatment, lung metastasectomy for RCC remains the reference curative treatment while stereotactic body radiotherapy (SBRT) is another option and has shown better local control than conventional fractionated radiotherapy [10]. RCC is considered less radioresponsive than other histologies.

The aim of this study was to evaluate results of RFA of lung metastases from kidney cancer in terms of local ablation efficacy, overall survival (OS), disease-free survival (DFS) and systemic treatment-free survival (STFS), and to define the factors associated with better outcomes.

## Methods

The aim of this study was to investigate local efficacy (absence of local recurrence), survivals and occurrence of complications after local treatment by RFA for RCC lung metastases treated over time at our institution, as well as the time without systemic treatment in order to report a potential effect of RFA on systemic treatment sparing. We also analyse factors associated with worse overall survival (OS), disease-free survival (DFS) and pulmonary progression-free (PPFS) survival.

This retrospective study included 53 patients (100 metastasis) treated in 65 sessions over 11 years at our large tertiary care centre. Patients with metastatic RCC (mRCC) were treated consecutively by RFA and registered in a database. Death notification or last patient follow-up news was obtained for all patients. This study was approved by the Institutional Review Board (Clinical Research Committee Institut Bergonié).

The decision to perform RFA was made after multidisciplinary discussion between radiologists, medical oncologists, radiation oncologists and surgeons. The radiologist informed the patient of the risks, possible complications, and benefits of the procedure. Limitations of and contraindications to RFA were represented mainly by the location of the tumor (< 1 cm from the hilum) and by the size of the tumor (> 40 mm). Pulmonary lesions with characteristics of new or enlarging metastases in patients with known RCC were considered as sufficient for diagnosis of lung metastases without recourse to biopsy. Biopsies were performed in uncertain cases.

Patients either had no extra-pulmonary metastases, or if extra-pulmonary metastases were present, they were controlled by previous surgery or radiotherapy.

### Radiofrequency ablation technique and follow-up

RFA was performed under helical CT guidance and under general anaesthesia. RFA used multiline expendable electrodes (LeVeen; Boston Scientific), with 3-, 3.5- or 4-cm array diameter when fully expanded.

Patients were followed up with whole-body CT imaging with intravenous contrast administration at 3, 6, 9 and 12 months and then every 6 months. Stability or any decrease in size was considered as complete treatment. An incomplete treatment was defined as regrowth, enlargement, or appearance of any irregular, nodular, or eccentric focus at the margin of the ablation zone by imaging. When a local failure was depicted, the patient file was reviewed by the multidisciplinary tumor group to determine the best option.

### Complications

All treatment-related complications were categorized in accordance with the standardized Common Terminology Criteria for Adverse Events (CTCAE), version 4.0, of the National Cancer Institute.

### Statistical methods

Median follow-up time was calculated by the inverse Kaplan-Meier technique and survivals by the Kaplan-Meier technique estimate rate were reported with their 95%CI. OS was calculated as the time between RFA and the date of death or last news.

DFS was calculated as the time between RFA and recurrence. Recurrences in the ablation zone, pulmonary out of the ablation zone and extra-pulmonary were considered as events. Patients alive or dead without events were censored. In PPFS any new lung tumor either distant or a recurrence on RFA treated lesions was considered. PPFS was calculated as the time between RFA and any pulmonary recurrence, patients alive or dead without pulmonary events were censored. Local recurrence-free survival (LRFS) was defined as the time between RFA and any recurrence on treated lesions, patients without any evolution of treated lesions were censored. The local primary success rate (local efficacy) was defined as the percentage of tumors that were successfully eradicated following the initial procedure or a defined course of treatment.

Free survival without systemic treatment (STFS) was defined as the time interval between RFA and the resuming ST or death without resuming the ST. Patients alive without the resumption of systemic treatment were censored at the date of last news.

We analysed: sex, age at RFA (≤/> 60 years), time interval between primary tumor and metastasis (concomitant or delayed), extra-thoracic metastases (yes/no), TNM classification (≤/> 2), Fuhrman grade (≤/> 2), size of the largest pulmonary lesion (≤/> 20 mm), and number of locations (1, 2, ≥3) as factors potentially associated with OS, DFS, PPFS and STFS with probability of Kaplan-Meier and log-rank test. All factors tested in univariate analysis were included in a multivariable backwards stepwise Cox manually regression model after verification of the proportionality hypothesis with the Shoendfeld test. SAS software, version 9.4, was used.

## Results

### Population characteristics

Thirty-four men and 19 women were included (Table 1), all of them treated with prior nephrectomy. One hundred metastases were treated by RFA. Median follow-up time was 61 months (interquartile range 90–34). Median number of metastases treated per patient was two (interquartile range 1–3). Median metastasis size was 12 mm (interquartile range 8 – 19 mm). Median time between primary tumor and first metastasis was 22 months (interquartile range 7–53 months).

**Table 1:** Population (*n* = 53) and Tumor (*n* = 100) characteristics

Characteristic	N (%)
Patient Sex	
Male	34 (64.2)
Female	19 (35.9)
Patient age at diagnostic (years)	
Median (interquartile range)	61 (20–84)
Patient age at Radiofrequency ablation treatment (RFA) (years)	
Median (interquartile range)	67 (29–86)
Treatment before RFA (per patient)	28 (53)
Interferon	18 (34)
Sunitinib	9 (17)
Sorafenib	1 (2)
Primary histology (per patient)	
Clear cell carcinoma	48 (90)
Papillary carcinoma	*1 (2)*
Mucinous tubular renal cell carcinoma RCC missing information	1 (2)1 (2)
Sarcomatoid component (10%)	2 (4)
T staging (per patient)	
T1 – T2	19 (36)
T3 – T4	25 (47)
Unknown	9 (17)
Tumor size in mm (per lesion)	
< 10	32 (32)
10 < x < 20	43 (43)
20 < x < 30	15 (15)
≥ 30	9 (9)
missing	1 (1)
Time to metastasis (months)	
Median (interquartile range)	22 (7–53)
Number of metastases (per patient)	
1	25 (47)
2	11 (21)
3	9 (17)
4–6	8 (15)
Number of RFA treatments (per patient)	
1	41 (77)
2	12 (23)
Fuhrman grade (per patient)	
1	1 (2)
2	9 (17)
3	26 (49)
4	5 (9)
Unknown	2 (3)
Time to recurrence (all types, per patient)	
≤ 12 months	20 (38)
> 12 months	33 (62)
Concomitant metastases (per patient)	
Concomitant	10 (19)
Delayed	43 (81)

### Recurrence and tumor progression

Overall, 13 (25%) patients with lung progression received a secondary RFA.

Nine local recurrences for nine patients occurred. The local efficacy percentage was 91% by lesion and 83% by patient. Seven of the nine local recurrences received a successful repeat ablation and achieved secondary local control (98%). The remaining two were not able to receive secondary ablation due to extrapulmonary progressive disease (mediastinal nodal involvement).

One and 3- year LRFS were 93.8% (95%CI 82.0–97.9) and 82.6% (95%CI 66.0–91.6) respectively.

### Survivals (Fig. 1)

Median follow-up time was 60.8 months (95% confidence interval [CI] 47.7–82.7). Median OS was not reached. OS at 1, 3 and 5 years was 94.0% (95%CI [82.6–98.0]), 74.5% (95%CI [58.5–85.1]) and 61.8% (95%CI [44.0–75.3]), respectively. Median DFS was 9.9 months (95%CI [6–16.4]) with DFS at 1 and 3 years of 40.3% (95%CI [27.0–53.3]) and 18.0% (95%CI [8.6–30.1]) respectively. Median PPFS was 21.7 months (95%CI [10.1–34.4]) with PPFS at 1 and 3 years of 58.9% (95%CI [44.1–70.93]) and 35.2% (95%CI [21.6–49.1]) respectively. Forty-two patients presented with progression in a median time interval of 8.5 months [interquartile range 3.9–20.8]. Of them, 24 were subsequently ST-free in a median time interval of 14.1 months [Interquartile range 6.1–24.8]. Median STFS was 28.3 months (95% CI [17.8–83.1]).

**Fig. 1:**
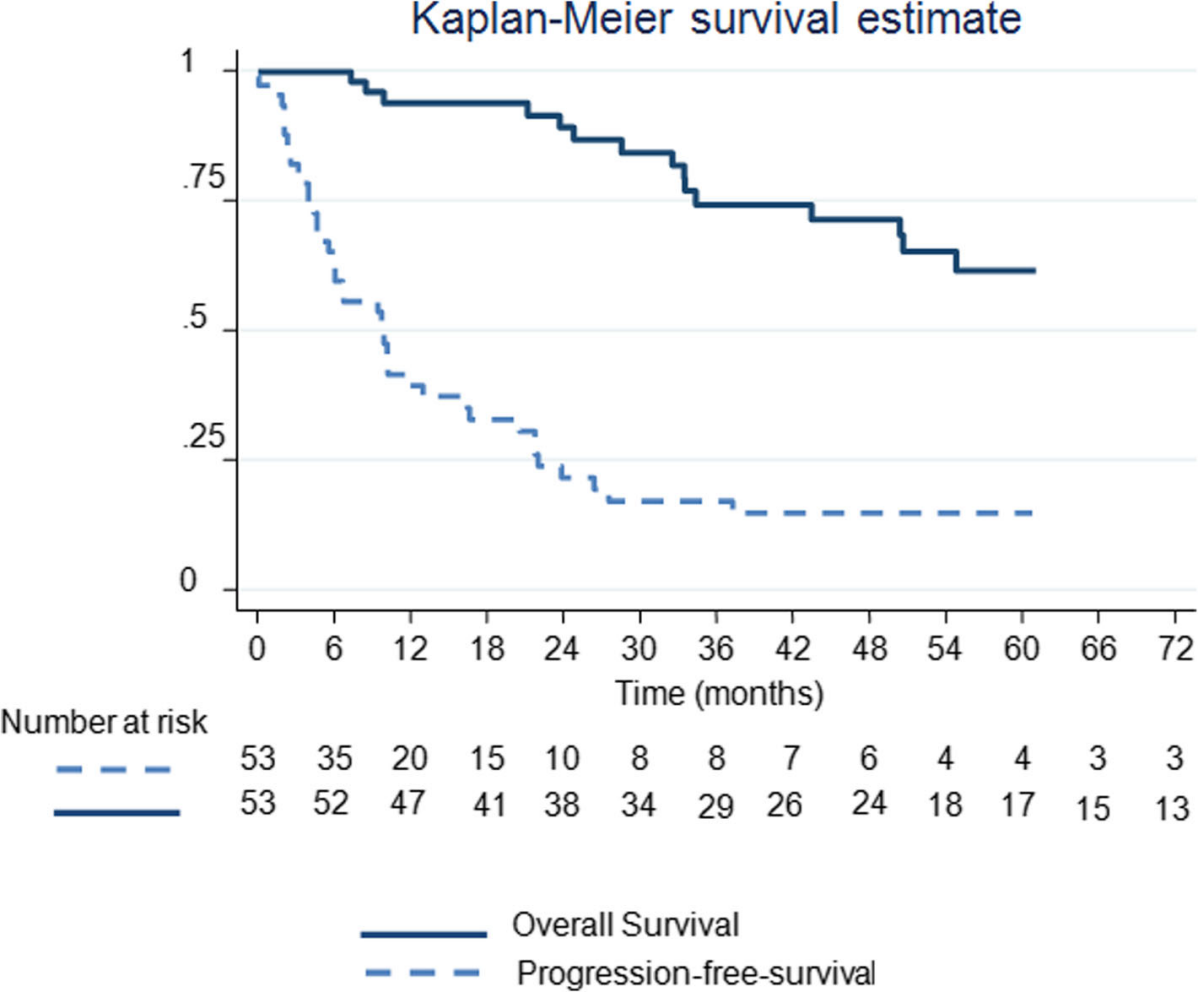
Kaplan-Meier Analysis of overall and disease-free survivals of 53 patients with renal cell carcinoma lung metastases treated by radiofrequency ablation

### Complications (Table 2)

Immediate complications occurred in 49 (75.4%) of the 65 procedures, some were only image findings without clinical complaints. Pneumothorax was the most frequent and expected complication, and 26 of 39 pneumothoraces were drained. The chest tube when necessary was removed after 1 or 2 days in the absence of recurrent pneumothorax excepted in one patient in whom a prolonged chest tube drainage was needed (8 days hospitalization). In 13 of the procedures, the pneumothorax was minor and no treatment was required.

**Table 2:** Complications and image findings^a^

	Number of procedures
Pleural effusion	32
Pneumothorax	39 (26 drained)
Infectious pneumopathies	4
Pain	19

^a^Only 3 were Grade 3–4 (pneumothorax requiring prolonged hospitalization; pain limiting daily activities; and a diaphragmatic hernia)

Only 3 complications were Grade 3–4 (pneumothorax requiring prolonged hospitalization; pain limiting daily activities; and a diaphragmatic hernia) (previously described) [11]. No procedural-related deaths were reported. The median duration of hospitalization was 3 days (range 1–8).

### Prognostic factors

At univariate analysis, tumoral staging of TNM classification of the primary RCC (*P* = 0.031) and largest pulmonary metastasis size > 20 mm (*P* = 0.019) were significant factors for OS. All factors tested in univariate analysis were included in a multivariable model and only T3/T4 TNM classification was identified as significantly associated with poorer OS (HR 3.55, 95%CI 1.05–12.1, *P* = 0.042) (Table 3).

**Table 3:** Factors associated with overall, disease-free, pulmonary progression-free, and systemic treatment free survivals after radiofrequency ablation of lung metastases from renal cell carcinoma (n = 53)

	Overall Survival		Pulmonary Progression-free survival		Systemic Treatment-free survival		Disease-free survival	
	Univariate	Multivariable	Univariate	Multivariable	Univariate	Multivariable	Univariate	Multivariable
	*P (Log-Rank)*	*HR (95%CI)*	*P*	*HR (95%CI)*	*P*	*P*		*P*
Sex								
Male	0.94	–		0.31	0.57	–	0.53	–
Female				2.7 (1.04–7.2) *(P = 0.042)*				
Age at RFA								
≤ 60 years	0.67	–	0.44	–	0.66	–	0.94	–
< 60 years								
Concomitant metastases								
Yes	0.87	–	0.85	–	0.71	–	0.81	–
No								
Extra-thoracic metastases								
Yes	0.86	–	0.73	–	0.79	–	0.72	–
No								
T staging								
T1-T2	0.031	1 (Ref.)	0.024	1 (Ref.)	0.047	1 (Ref.)	0.07	–
T3-T4		3.55 (1.05–12.1) (*P = 0.04)*		3.5 (1.3–9.3) *(P = 0.011)*		2.3 (1–5.5) *(P = 0.053)*		
Fuhrman								
1–2	0.31	–	0.49	–	0.26	–	0.71	–
3–4								
Lung mets size								
≤ 20 mm		1 (Ref.)	0.73	–	0.17	–	0.39	–
> 20 mm	0.019	2.56 (0.88–7.5) (*P = ns)*						
Number of lung metastases								
1	0.69	–	0.007	1 (Ref.)	0.60	–	0.14	–
2				3.09 (1.1–8.7) *(P* = 0.032)				
3				3.4 (1.3–9) *(P* = 0.013)				

No associations were identified between DFS and potential factors in univariate analyses, neither in a multivariable analysis.

At univariate analysis, tumoral staging of TNM classification of the primary RCC was a significant factor for PPFS (*P* = 0.024), as was number of locations (*P* = 0.007). All factors tested in univariate analysis were included in a multivariable model and both a T3/4 classification (HR = 3.5, 95%CI [1.3–9.3], *P* = 0.048), multiple lung metastases (2 metastases, HR = 3.1, 95%CI [1.1–8.7], *P* = 0.032; 3 metastases, HR = 3.4, 95%CI [1.3–9.0], *P* = 0.013) and sex (women HR = 2.7, 95%CI [1.04–7.2], *P* = 0.042) were associated with significantly worse PPFS.

At univariate analysis, tumoral staging (T) of TNM classification of the primary RCC was the only significant factor for STFS (*P* = 0.0469). All factors tested in univariate analysis were included in a multivariable model and only T3/T4 TNM classification was identified as significantly associated with poorer STFS (HR 2.33, 95%CI 0.9–5.5, *P* = 0.053).

## Discussion

In this large study investigating survival after local treatment by RFA for mRCC, good estimated probability of OS of 94, 74.5 and 62% at 1, 3 and 5 years respectively were observed. These rates can be compared with survival rates reported in surgical series after lung metastasectomy for mRCC, with 5-year OS estimated between 30 and 60% [12–14]. An association between lung metastasectomy and survival was reported [13] but only for patients in the intermediate risk group of the MSKCC prognostic classification [15]. Outcomes after local treatment by RFA of lung metastases have been reported previously on two small series (9 and 15 patients treated with curative intent) [8, 9]. Local efficacy of 91% and 5-year DFS of 23% were observed [8]. Our local efficacy, with probability of survival estimated at 91% is comparable, as is the observed DFS rate of 18% at 3 years. Until now, the largest published series of lung metastases treated with RFA concerned 566 patients [16] with several primary tumors, including kidney cancer (12%). The median OS was 62 months and the 4-year local efficacy was 89%.

Local efficacy was evaluated on CT images. Immediately after RFA, the ablation zone appears larger than the original tumor, because it consists of both tumor and perilesional ground-glass opacity corresponding to ongoing necrosis beyond the tumor margins. The enlargement is also explained by consolidation, inflammation, and hemorrhage, after which the ablation area will not increase in size. Then a consolidation will occur during the first 3 months the ablation zone continuing to be larger, compared with the original tumor, but should be smaller relative to the early phase as a result of regressing parenchymal edema, inflammation, and hemorrhage.

Roughly at 3 months, the size of the ablation zone should be larger than the baseline tumor, and by 6 months, the size of the ablation zone should be the same or smaller than the tumor before ablation and in general stable from this period or decreases. After 3 months any increase or appearance of any irregular, nodular, or eccentric focus at the margin of the ablation zone by imaging should be considered suspicious for tumor recurrence. In [16] follow-up evaluation with CT depicted 50% of cases of local progression during the first year of follow-up. Indeed, among 86 local progressions in 1037 metastases treated with RFA, 54, 21, 5 and 2 were diagnosed during the first, second third and fourth or fifth year of follow-up; reported rates of local tumor progression were 5.9, 8.5, 10.2, and 11% at 1, 2, 3, and 4 years, respectively.

Regarding prognostic factors, advanced tumor stage (T3 or 4 of the TNM classification) of the primary RCC was associated with poorer OS, PPFS and STFS. While T-staging is an important prognostic factor for localized RCC [17], this result may reflect the low metastatic load of such a selected population (85% ≤3 metastases). Conversely, Fuhrman grade was not significant, most likely because of patient recruitment for RFA. Only one patient had a Fuhrman grade 1 tumor and five patients had Fuhrman grade 4. Tumor grade is known to be an independent risk factor for recurrence and for tumor migration. There is a low metastatic potential for RCC Fuhrman grade 1 and a higher risk of polymetastatic evolution for Fuhrman grade 4 [18].

Although tumor size of pulmonary metastases > 20 mm was associated with poorer OS in the univariate analyses, this was only of borderline statistical significance in the multivariable model (*P* = 0.08), probably due to limited statistical power. The presence of two or more metastases was associated with poorer PPFS. Similar findings have been noted in surgical series [3, 12]. This relationship may be explained by an association between the risk of occult disease and the number of metastases [19]. Oligometastasis status is an empiric model [20] describing a biological state before polymetastatic status and so with a limited propensity for metastases. Women were also associated with poorer PPFS.

Time to recurrence was not a significant prognostic factor in our study. While time to recurrence was a criterion of oligometatastic state and prognostic for survival, some publications have reported that synchronous metastases do not have poorer prognosis than metachronous metastases [3, 14].

In contrast to previous studies [21] in our series, the presence of extra-thoracic metastases was not associated with survival outcomes. However, even if local treatment is indicated especially in the case of oligometastatic patients, other patients may also receive local treatment for lung metastases. Even for RCC with slow evolution, aggressive metastases with poor prognosis may appear due to clonal diversity [22]. Local treatment of these aggressive metastases may contribute to cancer disease control.

Local treatment has to be considered with systemic treatment strategy. Systemic treatment sparing may offer some advantages, when delayed at initiation [23] or discontinued, both in selected patients. Management of mRCC aims to improve the patients’ quality of life (QoL) while trying to prolong their survival. Targeted agents or immune therapies have improved survival but raise issues relating to the long-term delivery, with cumulative toxicity [24] and/or cost. Moreover, treatment discontinuation may reverse resistance to targeted therapies. The occurrence of early progression “rebound effects” has been cited as an argument against treatment discontinuation, but these have only been reported for patients with intermediate or poor prognostic MSKCC group [25]. While from 1.6 to 9% of complete response (CR) is achieved using the most recent first line systemic treatment [5, 26], partial response (PR) is more frequent and suggests a place of local treatment to make patients disease-free. One advantage of RFA is the ability to repeat local treatment to obtain total tumor clearance from the lung through repeated procedures, and may then answer to the needs of systemic treatment sparing in selected patients: 1-delay treatment initiation, 2-help to achieve CR in order to discontinue systemic treatment in case of PR, 3-prolong off period of systemic treatment. The concept of oligo-recurrence relates to patients with 1–5 metastatic or recurrent lesions that can be cured by local therapy with controlled primary lesions [27]. This suggests that a rigorous, repeated and comparative imaging surveillance is warranted. In our series, 13 (25%) patients could benefit from a new procedure when it was possible for lung recurrence. We observed a low rate of complications (3% grade 3/4). Due to the risk of pneumothorax, lung RF ablation was not an outpatient procedure. RFA does not impair lung function [7], was easily reproducible and well accepted, avoiding some of the morbidity associated with a repeat thoracotomy. A retrospective study [28] investigated discontinuation of targeted therapy in mRCC who achieved CR with either targeted therapy alone or a combined approach of additional resection of residual metastases. The median time without targeted therapies was 7 months. Another retrospective study reported a median time from CR to relapse of 7.9 and 8.2 months with or without local treatment [29]. In our series, 42 patients presented with progression in a median time interval of 8.5 months. Of them 24 resumed ST in a median time interval of 14.1 months. Median STFS was 28.3 months. To repeat local treatment may offer durable CR, and is an alternative to the resuming of ST and may be proposed as long as possible in patients with low-volume metastatic disease. RFA is not limited by the number of lung metastases, since a patient with 23 lung metastases has been successfully treated with RFA over a 10-year period, without impending lung function [30]. Moreover, like with radiotherapy, there is increasing interest in the abscopal and immunologic effects following thermal ablation [31]. With the use of newer targeted agents and immunomodulatory agents, optimization of patient selection through sequencing and combining the various treatment options is challenging.

Nine of our patients underwent a previous thoracotomy before RFA for lung progression. As for liver metastases, it is possible to combine RFA and surgical procedures to treat lung metastases completely. It is however difficult to obtain local control for central metastases because of the heat-sink effect and surgery is a better alternative, allowing lymph node resection. RCC is at risk of lymph node involvement, with an estimated prevalence of around 30% [32], and a mediastinal location in half of the cases. Mediastinal and hilar lymph node metastases significantly correlate with decreased survival [33]. Systematic lymphadenectomy provides valuable information on staging and prognosis in patients with pulmonary metastasis. In these locations, stereotactic radiotherapy is also an option for non surgical patients [34].

This study has some limits, mainly due to its retrospective design and evolutions in practice over the 11 years of the study e.g. treatment as well as prognostic classifications. In particular obtaining the Heng classification [35] was not possible retrospectively. Prognosis has evolved over this time, due to the development of new systemic treatments (with 53% of our patients receiving previously-used drugs of immunotherapy or target therapy before RFA). It is difficult to separate the effects of local RFA and systemic treatment. That biopsies were not systematically carried out for histological confirmation is another limitation.

## Conclusions

In conclusion, RFA is an effective and safe treatment of lung metastases from RCC, and it is a therapeutic option for local treatment of patients with or without comorbidities. Combined or sequential systemic therapy and local treatment enables good survival with the possibility of complete remission, with the possibility of delaying the need for systemic treatment for a long time. RFA is a good alternative to surgery offering low-morbidity and the possibility to repeat local treatment to obtain total tumor clearance.

## Data Availability

The datasets used and/or analysed during the current study are available from the corresponding author on reasonable request.

## Abbreviations

DFS: Disease-free survival
OS: Overall survival
PPFS: Pulmonary progression-free survival
RCC: Renal cell carcinoma
RFA: Radiofrequency ablation
STFS: Systemic treatment-free survival
